# Crowned Dens Syndrome Masquerading as Meningitis

**DOI:** 10.7759/cureus.12678

**Published:** 2021-01-13

**Authors:** Abayomi O Bamgboje, Nirupa Mohandas

**Affiliations:** 1 Internal Medicine, New York City Health + Hospitals/Metropolitan, New York, USA; 2 Internal Medicine/Rheumatology, New York City Health + Hospitals/Metropolitan, New York, USA

**Keywords:** meningitis, crowned dens syndrome, nonsteroidal antiinflammatory drugs, fever and neck stiffness

## Abstract

The classic symptoms of meningismus, including fever, neck stiffness, and headache, should automatically trigger a prime differential of meningitis, but a close masquerader, albeit rare, is crowned dens syndrome. Herein, we report the case of a 71-year-old woman with clinical features of meningismus with elevated inflammatory biomarkers. However, computed tomography of the cervical spine revealed the presence of calcium deposits encircling the dens. Hence, an alternate diagnosis of crowned dens syndrome was considered. This was confirmed by the presence of similar pathology in other joints and the dramatic resolution of symptoms and inflammatory markers with the administration of nonsteroidal anti-inflammatory drugs.

## Introduction

Crowned dens syndrome (CDS) is a clinico-radiological syndrome characterized by the presence of calcification in the ligament encircling the odontoid process. It presents with a variable duration of fever, meningism, and an inflammatory syndrome [1]. These microcrystalline deposits most often consist of calcium pyrophosphate dehydrate (CPPD) crystals or hydroxyapatite crystals. Patients can be either asymptomatic or symptomatic, presenting with a clinical picture similar to meningitis. CDS is commoner in the elderly as the mean age at the time of onset was found to be seventy-three years in men and sixty-two years in women, according to Goto et al. [2]. Herein, we report the case of an older woman with osteoarthritis (OA) who presented with acute attacks of CDS. We have attempted to examine the complex relationship between OA and the pathogenesis of CDS.

## Case presentation

A 71-year-old woman with a history significant for systemic hypertension, hyperlipidemia, and OA presented with fever, flu-like symptoms, and arthralgia for two weeks. While cough and runny-nose resolved, she continued to have persistent fever, malaise, and weakness. These were severe enough to cause a partial limitation in her activities of daily living. She also noticed pain in her left shoulder, left knee, mid-back, and neck that was associated with neck stiffness, which made her preferentially turn her head to the right side. The patient denied experiencing headaches, photophobia, phonophobia, nausea, vomiting, visual impairment, and upper or lower extremity weakness. There was no history of intravenous drug use, recent travel, coronavirus disease 2019 (COVID-19) contact, or surgery.

On physical examination, she was alert and oriented to place, time, and person. Her heart rate was regular but fluctuated between 93 and 110 beats/min, and her blood pressure was 200/109 mmHg. A temperature of 101.7 F was recorded. Neurologic examination was unremarkable, except for mild neck stiffness when the neck was passively rotated from side to side, which was worse on the left side. Kernig and Brudzinski’s signs were negative. She was unable to raise her left upper and lower limbs because of severe aching pain in her left shoulder and knee, but the power in the right upper and lower extremities was 5/5. She had bilateral tenderness of the deltoid muscles and severe left knee tenderness with a severely reduced range of motion.

**Figure 1:**
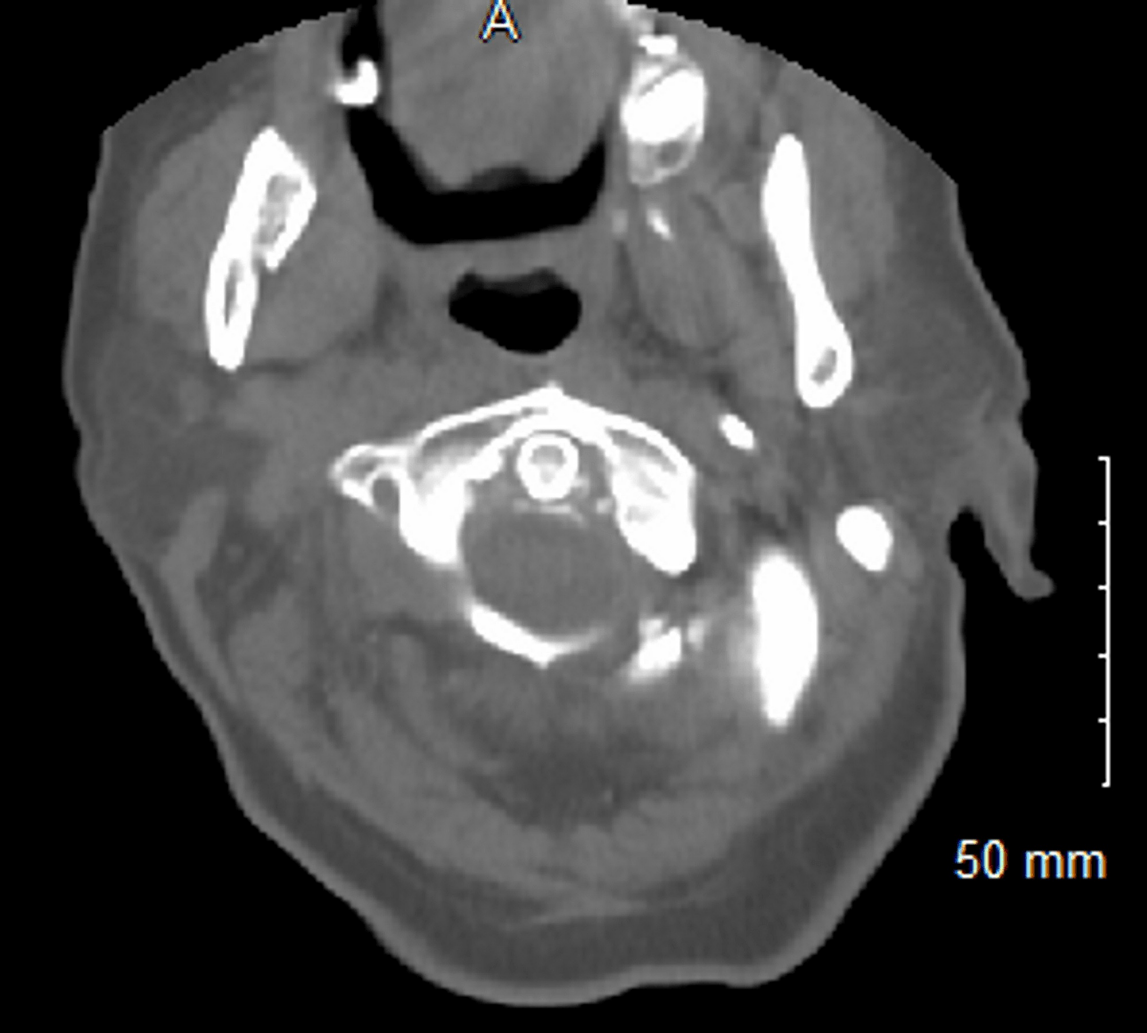
Cervical spine CT scan showing calcifications of the transverse and alar ligament (crowned dens syndrome)

**Figure 2:**
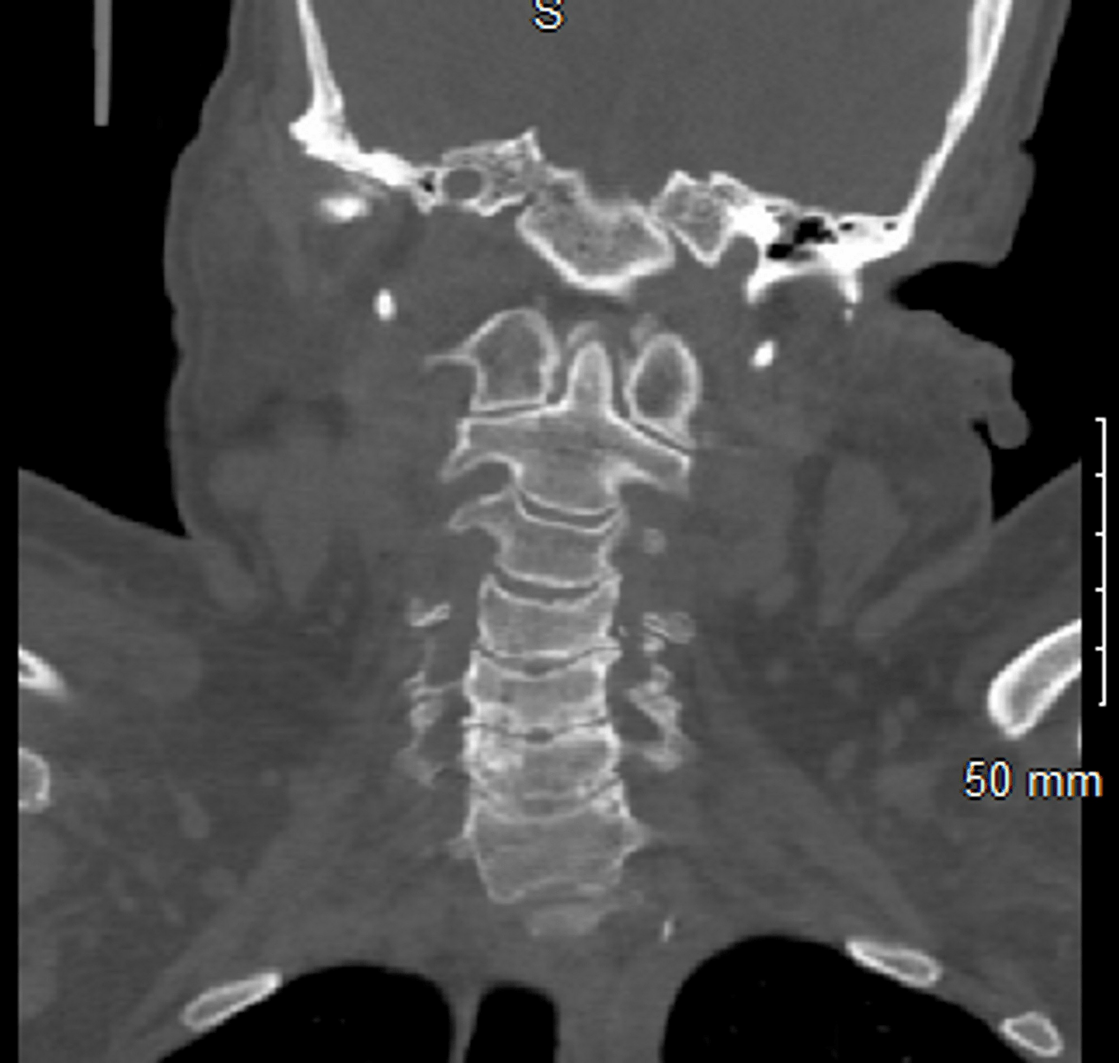
Cervical spine CT scan showing calcifications of the alar ligament (crowned dens syndrome)

**Figure 3:**
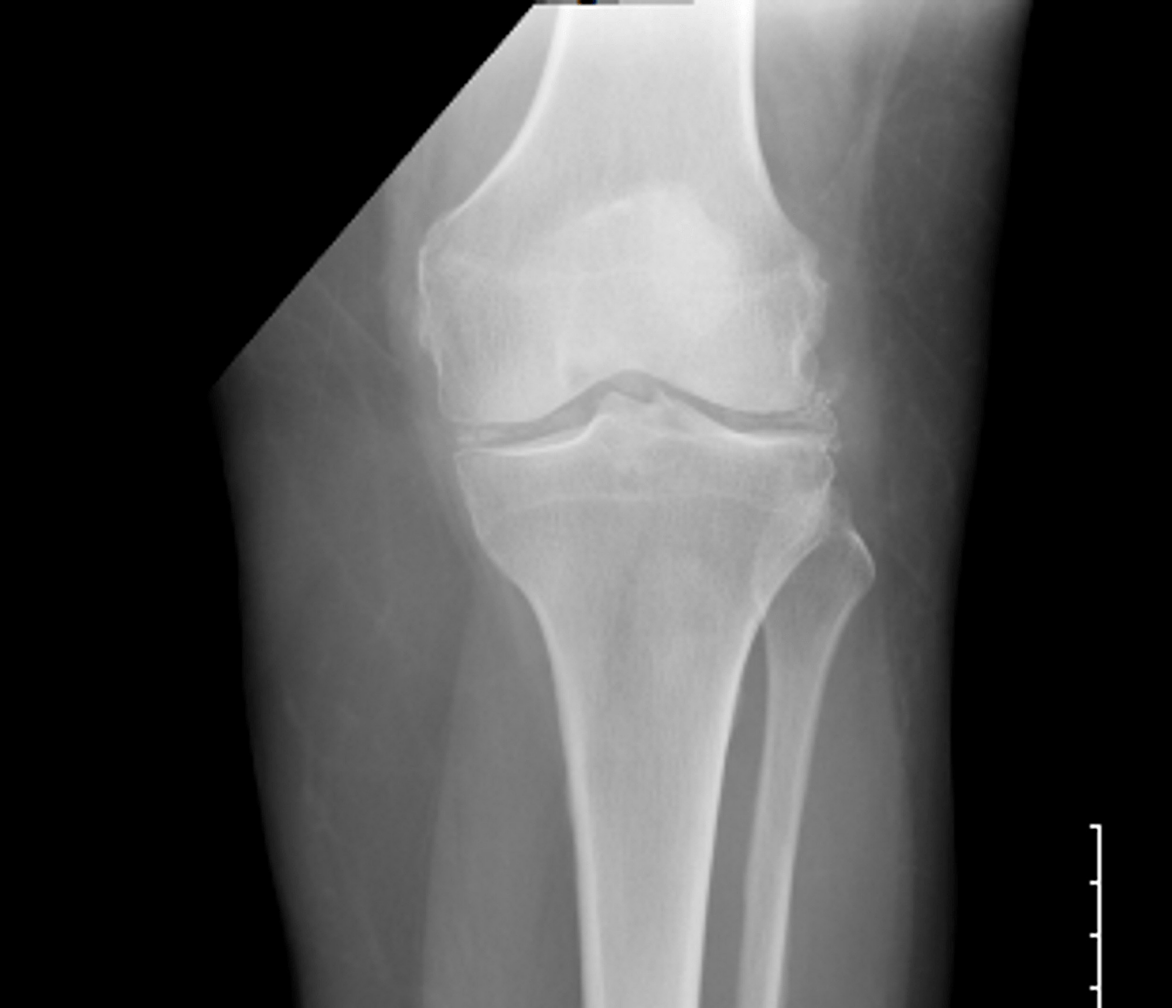
X-ray of the left knee joint showing chondrocalcinosis on the medial and lateral aspects

Blood tests revealed leukocytosis (23.75 × 10 9 cells/L); mildly reduced hemoglobin level (11.3 g/L); and elevated erythrocyte sedimentation rate (ESR: 125 mm/h), C-reactive protein (CRP: 480.66 ng/dL), and rheumatoid factor (17 IU/mL). The cyclic citrullinated peptide, thyroid-stimulating hormone, and anti-nuclear antibody levels were within the normal range. She had normal serum creatinine, low potassium, and low magnesium. She had elevated liver enzymes: alkaline phosphatase (257 IU/L), alanine transaminase (39 IU/L), and aspartate transaminase (40 IU/L). A rapid COVID-19 polymerase chain reaction test was negative. Creatinine kinase levels were normal, and blood cultures were negative. Computed tomography (CT) of the cervical spine (C-spine) showed calcification of the alar and transverse ligaments (Figures 1 and 2). However, brain CT revealed no acute intracranial abnormality. Hence, magnetic resonance imaging of the brain was performed, which revealed no acute intracranial hemorrhage, infarction, or abnormal intracranial contrast enhancement. A left knee joint radiograph showed chondrocalcinosis of the knee joint (Figure 3). CT of the lumbosacral spine showed left sacroiliitis. Urinalysis did not show any evidence of infection, likewise the chest radiograph. 

Infectious etiologies were considered as possible differential diagnoses, given the positive history of fever and leukocytosis, although no clear source could be identified despite extensive imaging and due to negative cultures. Meningitis was suspected because of the presence of fever and neck stiffness, but she had neck pain more than stiffness. There was no record of headache, scalp tenderness, altered mental status, sensitivity to light/sound, nausea, or vomiting. The presence of fever, arthralgia, and elevated inflammatory markers could have represented a rheumatologic process too.

Hence, we arrived at the diagnosis of CDS because of the finding of fever without the focus on the presence of clinico-radiological evidence of CDS and evidence of chondrocalcinosis in the left knee joint with left sacroiliitis that is known to be associated with CDS. Antibiotics were withheld after 24 hours of admission, and she was commenced on ibuprofen at a dose of 600 mg thrice daily. Her blood pressure was controlled with antihypertensives. In the next 24 hours, fever, neck pain, and neck stiffness resolved completely, and the left shoulder and left knee pain subsided significantly. The CRP levels had decreased to 186.56 ng/dL, and the white blood cell count had also dropped down to 9.98 × 109 cells/L within four days of starting ibuprofen. She had sustained clinical improvement with a total resolution of symptoms and was discharged 10 days after admission.

## Discussion

The presence of fever, headache, neck stiffness and inflammatory syndrome indicated meningitis, but CDS, which is usually under-diagnosed, should also be considered in this scenario [1, 3]. CDS is a rare disease, and its incidence is largely unknown. Out of the 2,023 patients who presented with neck pain in the study reported by Goto et al., 40 had CDS [2]. CDS is caused by microcrystalline deposits in the peri-odontoid articular and ligamentous structures, which typically look like a crown of calcium crystals encircling the dens [4]. Microcrystalline deposits usually consist of CPPD crystals and, less frequently, hydroxyapatite crystals. Patients can be asymptomatic or present with fever and neck pain [5]. The risk factors for CDS include age, OA, metabolic disorders (hypophosphatasia, hyperparathyroidism, hemochromatosis, hypomagnesemia), low cortical bone mineral density, and the presence of single nucleotide polymorphisms in ankylosis protein homolog. They are independent risk factors for CPPD and invariably CDS [6-8].

In our patient, the age, history of OA, and hypomagnesemia put her at high risk for CDS. Crystal formation is more common in such patients as articular calcification in OA is the consequence of uncontrolled ossification processes and a lopsided relationship between pro-mineralization factors and anti-mineralization factors. Chondrocytes in the osteoarticular cartilage of patients with OA have an increased rate of terminal differentiation and hypertrophy compared with normal chondrocytes. Matrix vesicles derived from these chondrocytes contain hydroxyapatite, which is released into the extracellular environment where calcium and phosphate ions form the core of new crystals [8].

The process by which CPP crystal-induced inflammation occurs is still unknown; however, studies have described a failed attempt at the digestion of insoluble crystals by macrophages causing ‘frustrated phagocytosis’ and lysosomal lysis in these macrophages, which leads to the release of reactive oxygen species, adenosine triphosphate (ATP),and potassium. This perturbation of cellular homeostasis causes oligomerization of nucleotide-binding domain, leucine-rich repeat family, pyrin domain containing 3 (NLRP3), and the recruitment of several apoptosis-associated speck-like proteins containing a caspase activation and recruitment domains (ASC; CARD) that combines with NLRP3 to form ASC specks. The NLRP3-ASC complex facilitates the recruitment of caspase 1, which undergoes autoactivation and, in turn, cleaves pro-interleukin-1β (pro-IL-1β) into its active form. Thus, IL-1β exacerbates inflammation by promoting cytokine and chemokine production from adjacent cells, endothelial cell activation, prostaglandin E2 production, and neutrophil recruitment [9, 10].

As seen in our patient, CDS can mimic and lead to an incorrect diagnosis of meningitis; when neck stiffness is associated with pain in the shoulder girdle and jaw claudication, polymyalgia rheumatica (PMR) and/or giant cell arteritis (GCA) may also be close differentials. CT scan focusing on C1/C2 is the gold standard for diagnosis [11]. This also helps delineate the anatomical subtypes of CDS [12]. The diagnosis of CDS is thus based on the association of clinico-radiological signs with a therapeutic response: acute attacks of neck stiffness, fever, joint pain with a biological inflammatory syndrome, radiological evidence of periodontal calcifications of the retro-odontoid ligament, and significant therapeutic response to nonsteroidal anti-inflammatory drugs (NSAIDs) or colchicine [4, 13, 14].

Although no medication exists that causes lysis and removal of calcium pyrophosphate (CPP) crystals from the joints in patients with CDS, these patients respond dramatically to NSAIDs, systemic corticosteroid treatment, or colchicine treatment [5, 15]. Systemic IL-1β inhibitors that target the pathogenesis of the disease have been reported to be beneficial for patients with acute CPP crystal arthritis, but this has not been tried in patients with CDS. Our patient had a resolution of symptoms and a decline in the inflammatory markers within four days of commencement of NSAIDs [16].

## Conclusions

A high index of suspicion, a thorough clinical examination, a functionally precise analysis of the CT of the C1-2 spine, and adjunctive X-ray images of the large joints in patients with severely elevated inflammatory markers and meningismus may obviate certain investigations and rule out differential diagnoses. Given that calcium deposits in the neck joints can also occur in other joints of the body, it may be necessary to look at the X-ray of large joints to further support the diagnosis of CDS. The resolution of these features with nonsteroidal antiinflammatory drugs confirmed the diagnosis of CDS.
